# The effect of vitamin D deficiency on the RANKL/OPG ratio in rats

**DOI:** 10.1016/j.jobcr.2022.02.004

**Published:** 2022-02-22

**Authors:** Rawan M. Khalaf, Abdullazez A. Almudhi

**Affiliations:** a Department of Pediatric Dentistry and Orthodontics, College of Dentistry, King Saud University, Graduate Studies and Scientific Research, P.O.Box 60169-15, Riyadh, 11545, Saudi Arabia; b Division of Orthodontics, Department of Pediatric Dentistry and Orthodontics, College of Dentistry, Graduate Studies and Scientific Research, P.O.Box 60169-15, Riyadh, 11545, Saudi Arabia

**Keywords:** Vitamin D deficiency, RANKL, OPG, RANKL/OPG ratio, PTH, Calcium, Phosphorus, ELISA

## Abstract

The aim of this study was to determine the effect of vitamin D deficiency on the RANKL/OPG ((Receptor Activator of Nuclear Factor Kappa B Ligand/Osteoprotegerin) ratio in the serum blood levels. Sixteen Wistar rats were divided into 2 groups: a control group (C) and an experimental group (E). The group C comprised of rats with average vitamin D levels, while vitamin D deficiency was induced in those of group E. A test period of 21 days was employed wherein two serum blood samples were obtained, at the baseline (day 0) and at day 21. Changes in RANKL andOPG levels were measured using Enzyme-linked immunosorbent assay (ELISA) kits. A statistically significant decrease of serum RANKL concentration and RANKL/OPG ratio on day 21 was seen in the experimental group compared to the control group. The serum RANKL levels and RANKL/OPG ratio in rats, were negatively affected by the deficiency of vitamin D.

## Graphical abstract

**Figure:**
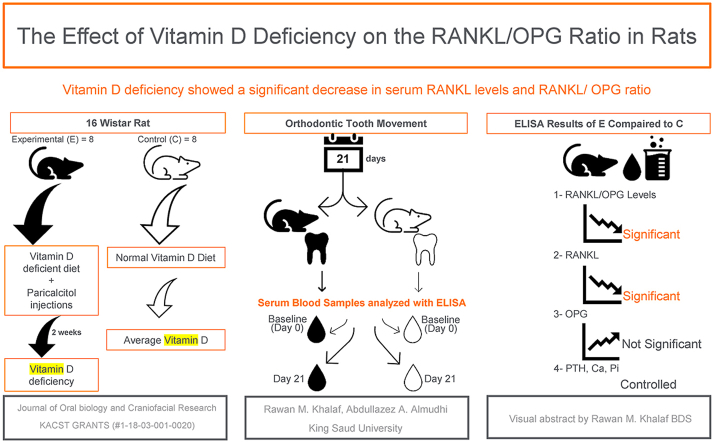

## 1 Introduction

One of the most common undiagnosed health conditions the world over, is vitamin D deficiency. Vitamin D has an important role in the regulation of bone metabolism.^1^ Bone metabolism depends on the equilibrium between vitamin D, calcium (Ca), calcitonin, and parathyroid hormone (PTH) levels.^2^

Vitamin D regulates the serum levels of Ca and phosphorus (Pi) by promoting their intestinal absorption and reabsorption in the kidneys. Furthermore, it promotes bone deposition and inhibits the release of PTH from the parathyroid glands, which in turn increases the concentration of Ca in the blood.^1^

Calcitonin is another hormone secreted by the thyroid gland and stimulated by the rise in calcium concentration in the blood, thus protecting the body against the development of hypercalcemia. Calcitonin is known to stimulate vitamin D,^3^ yet reduce root resorption, osteoclasts, and orthodontic tooth movement (OTM) distance in rats.^4^

When there is an inhibition of intestinal Ca absorption because of vitamin D deficiency, it leads to hyperparathyroidism, which stimulates bone resorption and loss.^5^

It has been found that there is an association between vitamin D levels and bone metabolism.^6^

Nimeri et al. reported that bone remodeling and OTM are controlled by some essential molecules, including vitamin D and RANKL/OPG system, which play a role in both physiologic and pathologic bone metabolism. RANKL binds to RANK on osteoclasts and accelerates OTM by osteoclastogenesis, while OPG is a decoy that inhibits RANKL from binding to osteoclasts. They stated that bone remodeling depends on the balance between the RANKL/OPG system, wherein the lower the RANKL/OPG ratio in serum blood, the more is the inhibition of OTM.^7^ RANKL/OPG ratio is vital for both skeletal integrity and bone mass. This ratio is considered to better reflect environmental signals of bone remodeling than the levels of each of these factors individually, where a high ratio level indicates bone resorption and a low level indicates bone formation.^8^^,^^9^

Vitamin D administration increases OTM rate by balancing bone remodeling.^10^ A study reported that vitamin D caused a spike in osteoclast number on day 14, after administration, and thereafter returned to baseline values. They also reported a significant increase in bone formation at the resorbed sites after OTM.^11^ Vitamin D increases the expression of RANKL and, therefore, a higher resorption rate.^12^ This was demonstrated when the administration of vitamin D in rats showed an increased resorption rate and remodeling, subsequently increasing the rate of tooth movement rate.^1^^,^^10^^,^^11^^,^^13^^,^^14^ Alongside its function as an activator for bone remodeling, vitamin D also enhances osteoblast performance coupled with reducing bone resorption.^14^

Therefore, the aim of this study was to evaluate the effect of vitamin D deficiency on the RANKL/OPG ratio in the serum blood levels, along with Ca, Pi, and PTH, and to ascertain the association between them.

## 2 Material and methods

### 2.1 Experimental animals

The study used sixteen, 300–330g weighing male Wistar rats, after obtaining an ethical clearance from the University. The sample power was calculated using the G power sample power calculator (Universtat Keil) using protocols for the calculation of power in animal experiments.^15^ It was estimated that for an effect size of 0.8 and power of 0.95 would need 8 rats per group to perform a paired *t*-test.

The cages that stored the rats were held in a suitable and quiet environment. They were constructed of type IV transparent polycarbonate, topped with metal mesh lid. The cages were distinguished into two categories: Group C, which included normal rats, and Group E, which had the experimental vitamin D-induced rats. The rats were disbursed into groups of three or two, and randomly assigned into their three-rat capacity cages. The rats experienced normal housing conditions; the holding cabinet held at room temperature (24–25 °C), relative humidity (55°), and a 12-h light-dark cycle. They were sheltered with abundance of food, and distilled water changed twice weekly. The experiment allowed the acclimation of the rats to the new environment of the facility, before commencing testing. Throughout the study, the animals were evaluated weekly for weight change.

All the animals were anesthetized with an intraperitoneal injection of Ketamine (Tekam 50 mg/ml, Hikma Farmaceutica Inc., Portugal) and Xylazine (Chanazine 20 mg/ml, Chanelle Pharmaceuticals Ltd Co., Ireland), as a dilution of 4 ml of Ketamine mixed with 1 ml of Xylazine and 1 ml of normal saline to get a dose of 0.2 ml for every 100 g/bodyweight of a single rat. In addition to Sevorane, an inhalation anesthetic (AbbVie Ltd. Abbott, Aesica Queenborough, UK) to complete the various procedures.^16^

### 2.2 Vitamin D deficiency induction in the experimental group

A period of 2 weeks was sufficient for vitamin D deficiency to be established in Group E, following the protocol of a previously introduced technique by Stavenuiter et al.,^17^ with an addition of one week of inactivity for observation before starting OTM. Maintenance of other conditions according to the protocol was performed with overthrowing the induction of vitamin D deficiency.

### 2.3 Blood sample collection protocol

Blood samples were collected on day zero and on day 21. For blood sample collection, the retro orbital technique (also referred to as peri-orbital, posterior-orbital and orbital venous sinus bleeding) to withdraw blood was used. Caution was used with providing sufficient time for recovery, where a large blood volume was necessary for our pre-experimental analysis (up to 0.2 ml or no more than 10% of body weight) under inhalation anesthesia with Sevorane. To harvest blood, a capillary pipette was inserted medially, laterally, or dorsally to the eyeball, blood is allowed to flow by capillary action into a serum separator tube with gel. The rats were monitored both pre and post-operatively and the technique provided quality samples of sufficient volume for analysis without causing injury to the animals.^18^

Samples were centrifuged at 2500 round per minute (RPM), and the plasma blood samples were obtained.

### 2.4 Phosphorus, calcium, PTH, RANKL, and OPG assessment

The levels of these variables were determined in the serum blood samples collected at baseline and just prior to sacrificing the animals, for both C and E groups using ELISA kits and Colorimetric Assays (Table 1).

**Table 1:** Assay Kits used in the study.

Name of Kit	Product Code	Purpose	Manufacturer
Phosphorus Colorimetric Assay Kit (Phospho-molybdate method)	E-BC-K245	Serum Phosphorus detection	Elabscience biotechnology Inc, China
Calcium Assay Kit, (With standard)	E-BC-K103	Serum Calcium detection	Elabscience biotechnology Inc, China
Rat I-PTH (Intact Parathormone) ELISA Kit	E-EL-R0535 96T	Serum PTH detection	Elabscience biotechnology Inc, China
Rat RANKL (Receptor Activator of Nuclear Factor Kappa B Ligand) ELISA kit	E-EL-R0841 96T	Serum RANKL detection	Elabscience biotechnology Inc, China
Rat OPG (Osteoprotegerin) ELISA Kit	E-EL-R0050 96T	Serum OPG detection	Elabscience biotechnology Inc, China

**Phosphorus Colorimetric Assay Kit:** Inorganic phosphorus reacts with molybdic acid to produce phosphomolybdic acid, which was reduced to molybdenum blue through the action of reducing agent. The molybdenum blue has a maximum absorption peak at 660 nm (nm); thus, the phosphorus content can be calculated indirectly by measuring the Optical Dentisty (OD) value at 660 nm.

**Calcium Assay Kit**: Calcium ions in the sample bind to Methyl Thymol Blue (MTB) in alkaline solution and form a blue complex. Calcium content can be calculated by measuring the OD value at 610 nm.

**Fig. 1:**
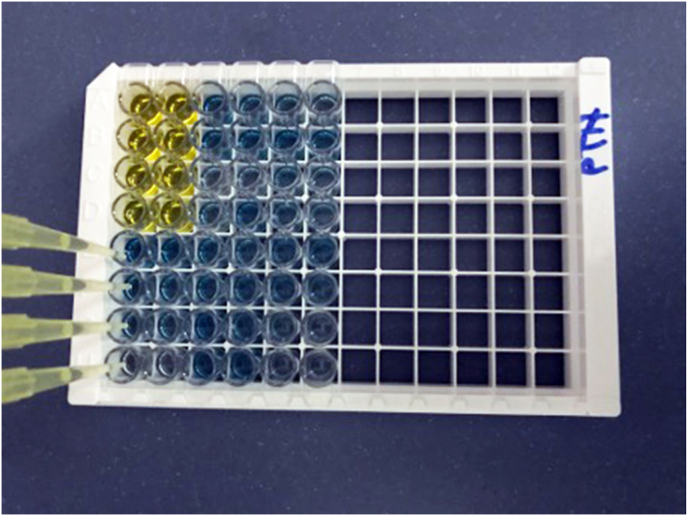
The enzyme-substrate reaction terminated by adding Stop Solution and appears yellow in color.

**ELISA Kits for Rat I-PTH, RANKL, and OPG:** The levels of RANKL, OPG, and IPTH were determined using individualized Commercial enzyme-linked immunosorbent assay (ELISA) kits according to the manufacturer's protocol. These kits use a Sandwich-ELISA method, where the ELISA plate provided in the kit was pre-coated with an antibody specific to rats. Standards or samples are added to appropriate micro-ELISA plate wells and combined with the specific antibody. The biotinylated detection antibodies specific for rat and Avidin-Horseradish Peroxidase (HRP) conjugate are added to each microplate well and incubated. After the incubation period, free components are washed away. The substrate reagent is added to each well; only those wells containing the rat variable, biotinylated detection antibody, and Avidin-HRP conjugate will appear blue (Fig. 1). The enzyme-substrate reaction will be terminated by adding Stop Solution, which renders it yellow in colour (Fig. 1).

**Fig. 2:**
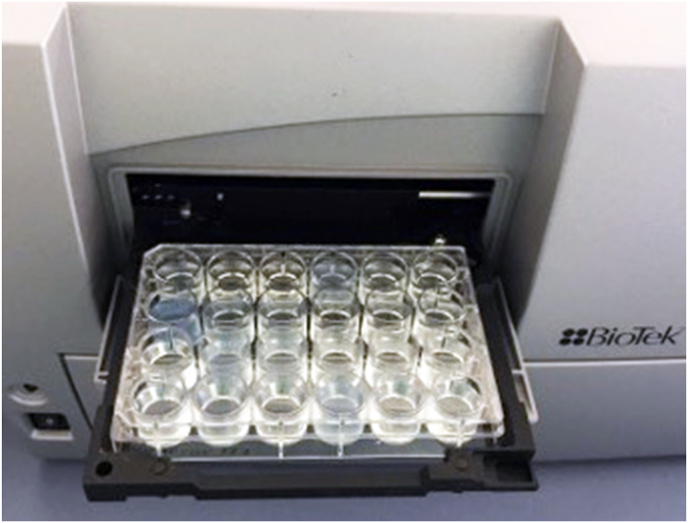
BioTek Synergy HT Microplate reader (Winooski, VA, USA).

The optical density (OD) can be measured with spectrophotometry at a wavelength of 450 ± 2 nm. The OD value is proportional to the concentration of Rat variable. The Rat variable concentration in samples can be calculated by comparing the OD of the samples with the standard curve.^19^ All ELISA variables were measured with BioTek Synergy HT Microplate reader (Winooski, VA, USA) (Fig. 2).

### 2.5 Statistical analysis

Normality of the sample was estimated using the Shapiro Wilk test and the mean concentrations of both vitamin D (p = 0.858), Calcium (p = 0.495), OPG (p = 0.374), Pi (p = 0.295), PTH1 (p = 0.356) and RANKL (p = 0.630) were found to be normally distributed. The difference in mean concentrations between groups was calculated using the independent samples *t*-test. The difference in means between time periods were calculated using the paired *t*-test separately for each group. The Pearson correlation was used to correlate between the total Vit D concentration OPG, RANKL and the Ratio of RANK/RANKL. The level of significance for all tests was set at p < 0.05 and all statistical analyses were performed using the SPSS ver. 25 data processing software (IBM-SPSS, Armonk NY, USA).

## 3 Results

When the baseline and day 21 levels of RANKL were compared between the test and experimental groups it was observed that there was no significant change in the control group (p = 0.189) but significant reduction in RANKL levels in the experimental group (p < 0.001). Similar findings were found for the OPG concentration and the ratio of RANKL to OPG where there was no significant difference in the control group but in the experimental group. Only the Vit-D levels showed significant reduction from baseline to day 21 in both control (p = 0.069) and the experimental group (p < 0.001) (Table 2).

**Table 2:** Differences in the test measures at baseline and Day 21.

Group	Parameter	Day 0		Day 21		t^a^	Sig
		Mean	SD	Mean	SD		
**Control**	**Total Vit. D (nmol/l)**	160.875	15.21688	176.125	18.48117	−2.148	0.069**
	**RANKL concentration, pg/mL**	1876.93	1699.26	1044.12	573.30	1.454	0.189
	**OPG concentration, pg/mL**	1436.56	233.33	1489.93	924.97	−0.175	0.866
	**Ratio of Rankl/OPG**	1.2425	1.10651	1.7719	2.76531	−0.051	0.666
**Experimental**	**Total Vit. D (nmol/l)**	51.6	8.64	20.9	3.75	14.916	<0.001**
	**RANKL concentration, pg/mL**	2871	1899.57	−1089.62	459.08	6.882	<0.001**
	**OPG concentration, pg/mL**	1181.66	176.86	1917.95	488.00	−3.586	0.009**
	**Ratio of Rankl/OPG**	2.344	1.41	−0.5812	0.229	6.423	<0.001**

** Differences significant at p < 0.05.

a Calculated using the paired *t*-test.

When the RANKL levels were compared between the experimental and control groups at day 21, it was observed that the experimental group showed a significantly greater decrease in RANKL levels (p < 0.001) and an increase in Vit D (p < 0.001). There was also a significant change in the ratio of RANKL/OPG (p = 0.047). There was, however, no significant difference in the OPG concentrations between groups. (Table 3).

**Table 3:** Comparison of test variables between test and control group at day 21.

	Group	Mean	Std. Deviation	Std. Error Mean	t^a^	Sig
**Total Vit. D (nmol/l)**	**Control**	**176.125**	**18.48117**	**6.53408**	**23.364**	0.00***
	**Experimental**	**20.3**	**3.7804**	**1.33657**		
**RANKL concentration, pg/mL**	**Control**	**1044.125**	**573.30924**	**202.69542**	**8.217**	0.00***
	**Experimental**	**−1089.625**	**459.08634**	**162.31153**		
**OPG concentration, pg/mL**	**Control**	**1489.93738**	**924.972972**	**327.027331**	**−1.158**	**0.266**
	**Experimental**	**1917.9585**	**488.006024**	**172.536184**		
**Ratio of Rankl/OPG**	**Control**	**1.7719**	**2.76531**	**0.97768**	**2.399**	0.047**
	**Experimental**	**−0.5812**	**0.22972**	**0.08122**		

** Differences significant at p < 0.05.

a Calculated using the independent samples *t*-test.

When the levels of Vit D, RANKL, OPG and ratio RANKL/OPG were correlated it was observed that there were no significant correlations between the readings in either the experimental or the control groups.

## 4 Discussion

For the purpose of the experiment, Wistar rats were used, which have many advantages as their periodontal tissue and anatomical structure are similar to those in humans. They are also relatively inexpensive, and it is easy to breed larger numbers of the animals. Rats' weight during the study was regularly monitored and recorded; with only the normal physiological increase in weight along with age seen.

To induce vitamin D deficiency, a method that was previously developed by Stavenuiter et al. was used, wherein, after completing the injections, an inactive period of one more week was observed, during which a vitamin D deficient diet was continued to ensure normal levels of PTH, Ca, and Pi.^17^

In this present study, PTH and Pi were controlled, and there was no significant change reported. Calcium, however, was significantly decreased in both groups on day 21; yet not enough to affect PTH and Pi levels.

The ELISA method was used for blood serum analysis; many investigators like Li et al. also used sandwich ELISA as a method for analysis of biochemical markers of bone turnover of total Rat RANKL and rat OPG in blood serum.^20^ However it should be noted that biochemical markers of bone from blood serum samples used for in vitro animal studies may differ from what may actually be seen in humans.^21^ Caution must be taken when applying the results from animal studies on humans; to eliminate any discrepancies, further in vivo investigations on humans are required.

RANKL and OPG have greatly enhanced the understanding of bone biology. RANKL is expressed by osteoblasts and their precursors and is necessary for osteoclastogenesis. It activates its receptor, ‘RANK’, expressed on osteoclasts, along with their precursors to promote osteoclast differentiation and activation. The effects of RANKL are blocked by OPG, which acts as a decoy receptor for RANKL. Therefore, the RANKL/OPG ratio is critical in establishing the balance between bone formation and resorption.^22^

In a study of periodontitis on male Wistar rats that had low doses of metformin (50 mg/kg), RANKL and OPG were visualized using a colorimetric-based detection kit; they found weak staining of RANKL (reduced) but strong staining of OPG, which indicated a decrease in bone resorption with reduction of osteoclasts number and an increase of osteoblasts.^23^

Sun et al. utilized a high dose of 1,25(OH)2D3 in rats; their study revealed significantly decreased bone volume in the experimental group with more bone resorbed than control animals with a change in RANKL/OPG ratio through having a stronger RANKL and weaker OPG expressions at all time durations.^24^

Their results were concurrent with the present study despite the different experimental model wherein vit D deficiency was induced.

Those previous studies support our findings, as RANKL concentration and RANKL/OPG ratio decreased significantly in the experimental group compared to the control group on day 21.

Erban et al. studied the effect of high doses of vitamin D on Rats bone marrow and suggested that this high dose would enhance bone formation and osteoblasts. However, the doses used in humans may not be high enough to show an early increase in bone formation.^14^

Rats orthodontic histological changes in the periodontal tissue and its mechanism are the same as in humans, but faster. Nevertheless, data derived from rat and animal findings must be interpreted and practiced with caution before conclusions are made for human conditions.^25^ Further evidence is needed to determine the vitamin D deficiency patients, who are seeking orthodontic treatment, which would suggest routine referrals for adjusting vitamin D to an optimal levels by supplementations, where patients and practitioners would anticipate a longer treatment time with a slower tooth movement if vitamin D was not normalized. It was advised that a dentist confirms with the concerned physician for the fitness of patients prior to undergoing OTM.^10^

The present study demonstrated the ability of vitamin D deficiency to reduce RANKL and RANKL/OPG ratio; these findings are to be confirmed with future studies using micro-CT and histological analysis with a more detailed microbiological level results that can be considered. It can be concluded that Wistar rats with induced Vitamin D deficiency showed a significant decrease in serum RANKL levels and RANKL/OPG ratio; however, there was no significant correlation between RANKL, OPG, and RANKL/OPG ratio.

## Funding

This funding was supported by 10.13039/501100004919King Abdulaziz City for Science and Technology (KACST) by the Research and Development Grants Program for National Research Institutions and Centers (GRANTS) for graduate students’ research program (#1-18-03-001-0020) in Riyadh- Kingdom of Saudi Arabia.

## Declaration of competing interest

The authors declare that there is no conflict of interest regarding the publication of this paper.
